# Preliminary grip strength data from an 8‐week pilot trial of creatine monohydrate supplementation in Alzheimer’s disease patients

**DOI:** 10.1002/alz.092602

**Published:** 2025-01-09

**Authors:** Aaron N. Smith, Debra K Sullivan, Emma Kelly, Tanu N Arora, Faith N Waitsman, Matthew K Taylor

**Affiliations:** ^1^ University of Kansas Medical Center, Kansas City, KS USA

## Abstract

**Background:**

Diminished muscle function and strength are linked to an increased risk and accelerated progression of Alzheimer’s disease (AD). Although these are direct consequences of the disease, recent preclinical evidence suggests decreased muscle function and strength may directly influence AD risk and progression via the muscle‐brain axis. Therefore, interventions to improve muscle strength may prevent functional decline related to AD. However, such specific interventions in AD are lacking. In this context, creatine monohydrate (CrM), a dietary supplement that boosts muscle strength, presents a promising intervention. This single‐armed pilot study explores the potential effects of an 8‐week creatine monohydrate supplementation on handgrip strength in AD.

**Method:**

Data from 16 participants with AD who completed a one‐arm, 8‐week trial examining the feasibility and preliminary efficacy of 20 g/day of CrM in AD were analyzed. Handgrip strength, a reliable measure of muscle strength, was assessed on the dominant hand at baseline and at the end of the study on a calibrated hand dynamometer, measured in kilograms (kg) of force. At baseline and 8 weeks, 3 measurements were taken; the highest value was selected. We conducted a paired t‐test to test for an 8‐week change in mean handgrip strength. Statistical analyses were performed using R (v. 4.3.2; R Foundation, Vienna, Austria). Statistical significance was set at p < 0.05.

**Result:**

Participants were 56% male with a mean age of 72.1 ± 6.4 years at baseline. The mean hand grip strength and body mass index (BMI) at baseline were 33.6 ± 11.6 kg and 25.4 ± 3.6 kg/m^2^, respectively. After 8 weeks of CrM supplementation, mean handgrip strength improved by 2.6 kg (36.1 ± 12.1 kg, p < 0.001). BMI did not change.

**Conclusion:**

In this study, 20 g/day of CrM supplementation for 8 weeks was associated with improved handgrip strength in individuals with AD. Our pilot data suggests that CrM may be valuable for maintaining or preventing AD‐related functional decline by improving muscle strength.

